# Immune infiltration and drug treatment response of angiogenesis-related LncRNA in lung adenocarcinoma

**DOI:** 10.1097/MD.0000000000042958

**Published:** 2025-07-04

**Authors:** Qing Lei, Biao-Feng Fan, Xi-Rui Zhu, Bo-Wen Cui, Jin-Xiang Kong, Zhong-Rui Ma, Xi-Qi Xie, Wei-Wei Wang

**Affiliations:** a Department of Thoracic Surgery, The Third Affiliated Hospital of Kunming Medical University, Yunnan Cancer Hospital, Yunnan Cancer Center, Xishan District, Kunming, China.

**Keywords:** angiogenesis, immune, long noncoding RNA, LUAD, treatment

## Abstract

**Background:**

Lung adenocarcinoma (LUAD) is one of the most lethal tumors and is characterized by high mortality and worse prognosis. Long noncoding RNA (lncRNA) plays an important function in tumor formation. Targeting tumor angiogenesis is a crucial cancer treatment strategy. So, this study aims to explore the effects of angiogenesis-related lncRNA (ARlncRNAs) signatures in the prediction of clinical prospects, immunotherapy, and their association with drug sensitivity.

**Methods:**

The Cancer Genome Atlas (TCGA) database was used to get the genetic and clinical information. Co-expression analysis and Cox regression analysis were used to create prognostic profiles. To assess and validate the model’s predictive efficacy, principal component analysis, survival analysis, and receiver operating characteristic curves were used. To enhance the model’s prediction, a nomogram of forecasts was created, and calibration curves were used. Last, we studied variations in tumor mutational burden, immune-related function, and antiangiogenic medication sensitivity between high- and low-risk cohorts.

**Results:**

We successfully constructed an angiogenesis-related prognostic signature for LUAD, including 6 lncRNAs (AL157388.1, AL590428.1, LINC02057, AC245041.1, AC068228.1, and AL365181.2). Independent predictive analysis, receiver operating characteristic curve, C-index, and nomogram diagnostic results showed that ARlncRNAs accurately predicted outcome and 1-, 3-, and 5-year overall survival. According to the analysis of the differences in immune-related pathways between high- and low-risk cohorts, the low-risk cohort had a more active immune function. An analysis of immune checkpoints showed that low-risk patients had higher expression of immune checkpoints, which means that low-risk LUAD patients had a more active immune function; these patients may benefit from checkpoint blocking immunotherapy. Screening for sensitive drugs by predicting the IC_50_ of antivascular drugs, the high-risk cohort has lower IC_50_ values and is less likely to be resistant than the low-risk cohort.

**Conclusion:**

Overall, the new predictive features constructed based on ARlncRNAs can effectively predict the outcome of patients and offer a fresh perspective for LUAD diagnosis and therapy.

## 1. Introduction

Lung cancer, originating from the bronchial mucosa or glands of the lungs, is one of the most common and deadly malignancies. Recent statistics indicate that approximately 2.2 million new cases of lung cancer are diagnosed annually worldwide, accounting for 11.4% of all malignancies.^[1,2]^ Among these, non-small cell lung cancer is the most common subtype, representing 80% to 90% of all lung cancers. Lung adenocarcinoma (LUAD), the predominant type of non-small cell lung cancer, has been showing a trend toward younger age of onset in recent years,^[3]^ and it has a continuing increase in the prevalence rate among current smokers and even nonsmokers.^[4]^ Treatment for lung cancer is stage-dependent: patients with early-stage disease can often be cured by surgical resection, while those with the locally advanced diseases require multimodal therapy, including chemotherapy, radiotherapy, and immunotherapy.^[5,6]^ LUAD continues to be one of the most aggressive and deadly tumor forms, despite several improvements in diagnostic and treatment approaches. Consequently, it is crucial to investigate more thorough clinical diagnosis techniques for LUAD and discover new powerful biomarkers and therapeutic approaches that can help predict and improve the clinical prognosis of LUAD patients.

Angiogenesis refers to the formation of new blood vessels from preexisting capillaries or small postcapillary venules.^[7]^ Tumor angiogenesis is consistently exceptional in growing tumors because they are required to deliver oxygen and nutrients through the circulatory system to survive and proliferate.^[8]^ During tumor progression, hypoxia can stimulate the production of various factors that promote angiogenesis, including growth factors, chemokines, extracellular matrix components, and integrins, which collectively enhanced tumor angiogenesis.^[9]^ In addition, angiogenesis may facilitate the entry of cancer cells into the bloodstream, contributing to hematogenous metastasis.^[10]^ During the last decades, accumulating evidence has established angiogenesis as a critical cancer biomarker, with its presence often correlating with poor prognosis in various solid tumors. The “angiogenic switch” is directed by signaling molecules,^[11]^ such as vascular endothelial growth factor (VEGF), which plays a pivotal role in inducing tumor angiogenesis. Besides these signaling factors, recent studies have also indicated that long noncoding RNA (LncRNA) is a possible factor driving tumor angiogenesis.^[12]^

LncRNAs are RNA molecules longer than 200 nucleotides that do not code for proteins. These molecules are involved in a wide range of biological processes, such as cell differentiation, apoptosis, and various pathophysiological processes.^[13,14]^ Numerous studies have shown that dysregulated LncRNAs play a significant role in the hallmarks of cancer, including metastasis, drug resistance, and angiogenesis.^[15]^ LncRNAs can influence oncogenic pathways either directly or indirectly by regulating tumor-associated cells or act as key regulators of tumor angiogenesis in combination with other RNA transcripts.^[16]^ However, there has been no systematic assessment of the features of angiogenesis-related LncRNAs (ARLncRNAs) in LUAD patients, and it is uncertain whether ARLncRNAs are helpful for predicting survival in LUAD patients. In this study, we established prognostic signatures of 6 ARLncRNAs (AL157388.1, AL590428.1, LINC02057, AC245041.1, AC068228.1 and AL365181.2) in LUAD patients, which were identified as critical prognostic factors and potential therapeutic targets.

## 2. Methods

### 2.1. Preparation of data and clinical information

LUAD RNA-seq transcriptome, clinical, and tumor mutation burden (TMB) data were obtained from the Cancer Genome Atlas (TCGA) database (https://portal.gdc.cancer.gov/). RNA-seq transcriptome expression data were subjected to normalization, and samples were excluded if they were missing from the clinical profile. To identify angiogenesis-related genes involved in the angiogenesis process, the Molecular Signature Database v7.4 was used. The tissue samples used in this experiment were obtained with the informed consent of the patients, provided by the Yunnan Cancer Hospital, and approved by the Ethics Committee of the Yunnan Cancer Hospital.

### 2.2. Identification of angiogenesis-related LncRNAs

To ensure biological relevance, LncRNAs with a median normalized expression of < 1 fragments per kilobase of exon per million fragments mapped across all samples were excluded. Pearson correlation (|Cor| > 0.4, *P* < .001) was then used to filter ARLncRNAs. The LUAD transcriptome data were compiled using Perl (Strawberry Perl 5.30), differentiating between mRNAs and LncRNAs. The expression of genes related to angiogenesis was extracted using the “limma” package in R (Rx64 4.1.0), and ARLncRNAs were further filtered based on common expression criteria, correlation and a Wilcoxon test.

### 2.3. ARLncRNA prognostic modeling and validation in LUAD

Tumor samples were randomly divided into training and testing cohorts at a ratio of 1:1, and between-cohort differences by chi-square test were compared in order to build the best LUAD ARLncRNA prediction prognostic model. The prognostic model was built using data from the training cohort, and model validation was performed using data from the test cohort and the total cohort. The potential ARLncRNAs were first identified using the R “survival,” “survminer,” and “glmnet” software packages. Next, the overfitting genes were reduced using the least absolute shrinkage and selection operator (LASSO), and the best risk prognostic model was created based on the findings of the multivariate Cox regression analysis. The following formula was used to determine each LUAD sample’s predictive risk score for ARLncRNAs:


$$\begin{array}{l} RiskScore=\sum inCoef(i)*Expr(i). \end{array}$$

Each LncRNA’s expression level is denoted as Expri,while its corresponding coefficient is represented as Coefi. LUAD samples were categorized into a high-cohort and a low-risk cohort based on the median risk score. In order to investigate the possible reciprocal regulatory link between them, the expression of ARLncRNAs involved in the risk model formulation was extracted and associated with genes relevant to angiogenesis. To evaluate survival differences between the high-risk and low-risk cohorts, we performed Kaplan–Meier survival analysis, generated risk score curves, and created survival status scatter plots for the training, test, and total cohorts using the R packages “survival” and “survminer.” Additionally, univariate and multivariate Cox regression analyses were conducted to determine whether this risk model could serve as an independent prognostic factor for LUAD. The predictive performance of the model was assessed by constructing a receiver operating characteristic (ROC) curve and calculating the area under the curve.

### 2.4. Construct a nomogram survival model

For the purpose of estimating the overall survival (OS) probabilities of LUAD patients at 1, 3 and 5 years, we were constructed a nomogram survival score chart including age, gender, staging, and risk score. Furthermore, we used calibration curves to evaluate the level of concurrence between actual outcomes and nomogram predictions. Using the “rms” package, the consistency index in R software was utilized to measure the predictive power and stability of the nomogram.

### 2.5. Principal component analysis (PCA)

PCA^[17]^ was used to compare the variations between high- and low-risk cohorts based on all gene sets, angiogenesis-related gene sets, ARLncRNA sets, and prognostic model ARLncRNA sets.

### 2.6. Enrichment of pathways in the risk prognosis signature

We used Gene Set Enrichment Analysis (GSEA) software (version 4.3.2) (http://www.gsea-msigdb.org/gsea/index.jsp) to conduct GSEA and identify pathways significantly enriched between the high-risk and low-risk cohorts. Pathways with *P* < .05 and false discovery rate < 0.25 were considered statistically significant. Data visualization was performed using the R packages “grid” “gridExtra” and “ggplot2.”

### 2.7. TMB

The LUAD mutation data was downloaded from the TCGA database and used to construct the TMB. In order to assess the prospective utility of TMB and compare the differences between the 2 cohorts, the LUAD sample was split into 2 cohorts based on the median TMB: a high TMB (H-TMB) cohort and a low-TMB (L-TMB) cohort.

### 2.8. Immunological correlation analysis and drug sensitivity analysis

The difference of immune-related function of LUAD patients was analyzed by “limma” and “GSVA” packages, and *P* < .05 was considered as statistically significant. The results were visualized using the “phatmap” package. The half-maximal inhibitory concentration (IC_50_) of chemotherapeutic agents was predicted using the “pRRophetic” “ggplot2” and “ggpubr” packages.^[18]^ Candidate agents were screened based on Pearson correlation coefficients, and differences in IC_50_ values between high- and low-risk cohorts were assessed using the Wilcoxon rank-sum test.

### 2.9. Quantitative real-time polymerase chain reaction (qRT-PCR)

Total RNA was extracted using TRIzol reagent (Invitrogen) and reverse transcribed into complementary DNA using PrimeScript RT Reagent Kit (Takara, China). SYBR Premix Ex Taq (Takara, China) was used to perform qRT-PCR according to the manufacturer’s instructions. Our primers were designed with reference to the previous study.^[19]^ Primer sequences were generated using Primer3 software. The primer sequences used in this study are listed in Supplementary S1, Supplemental Digital Content, https://links.lww.com/MD/P252. GAPDH was the endogenous control.

## 3. Results

### 3.1. Identification of ARLncRNAs in TCGA LUAD dataset

**Figure 1.**
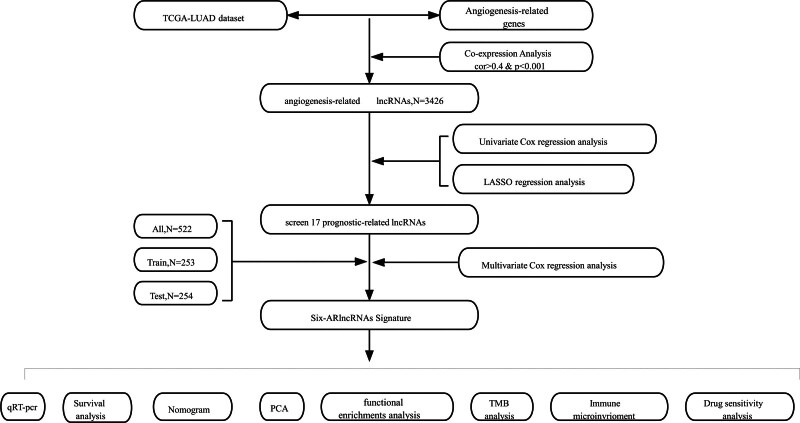
Study flowchart shows the process of constructing and assessing the 6 ARlncRNAs prognostic signature for lung adenocarcinoma. ARlncRNAs = angiogenesis-related LncRNAs.

**Figure 2.**
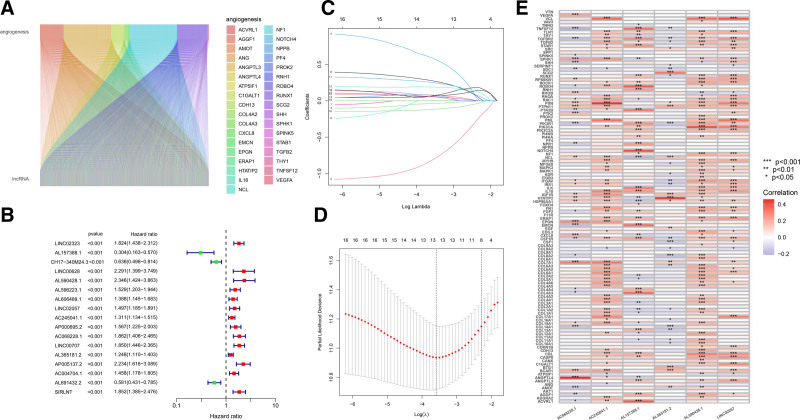
(A) Co-expression relationships between angiogenesis-related genes and ARLncRNAs; (B) forest plot of univariate Cox regression analysis; (C) the dynamic process of applying LASSO regression analysis to filter variables; (D) selection process of the optimal cross-validation parameter λ in the LASSO model; (E) correlation heatmap of ARLncRNAs with angiogenesis-related genes, red color indicates a positive correlation and blue color indicates negative correlation. ARlncRNAs = angiogenesis-related LncRNAs, LASSO = least absolute shrinkage and selection operator.

Figure 1 displayed a thorough flow chart of our investigation. The TCGA LUAD transcriptome dataset included 541 tumor samples and 59 normal samples. We used Perl to discriminate between mRNA and LncRNA. We ultimately obtained 3426 ARLncRNAs that were based on the expression of 119 angiogenic genes and the differential expression of LncRNAs in normal and tumor samples (Supplementary S2, Supplemental Digital Content, https://links.lww.com/MD/P253). Co-expression relationships were shown between angiogenesis-related genes and ARLncRNAs by using Sankey plots (Fig. 2A).

### 3.2. Construction and evaluation of the prognostic relationship between ARLncRNAs and LUAD

After excluding patients with a survival period of <30 days and those with lacking survival information, 522 LUAD patients took part in the development and validation of the ARLncRNA signature. These patients were randomly assigned into a training cohort (n = 253) and a test cohort (n = 254) at a 1:1 ratio. No statistically significant differences (*P* < .05) were observed between the 2 cohorts in clinical characteristics such as age, gender, and tumor stage. Additionally, when using the total dataset as the validation cohort, no significant differences were found in age (*P* = .2625), gender (*P* = .9671), tumor stage (*P* = .741), T classification (*P* = .7131), N classification (*P* = .4156), or M classification (*P* = .6681) (Supplementary S3, Supplemental Digital Content, https://links.lww.com/MD/P254).

We performed univariate Cox regression analysis (*P* < .001) on the TCGA dataset to identify 17 ARLncRNAs that were significantly associated with survival in LUAD patients (Fig. 2B). In order to prevent overfitting and to increase the precision and interpretability of prognostic features, overfitting genes were minimized using LASSO constraint parameters (Fig. 2C and D), and multifactorial Cox regression analysis was carried out to test 6 ARLncRNAs for the optimum risk prognostic model. we established a signature composed of 6 LncRNAs (AL157388.1, AL590428.1, LINC02057, AC245041.1, AC068228.1, and AL365181.2) were identified as potential prognostic biomarkers for LUAD, and based on the expression and coefficients of these 6 LncRNAs, multiple Cox regression calculation of risk scores for each patient in the training cohort. Risk score = [AL157388.1 expression × (-1.3780)] + [AL590428.1 expression × (0.7306)} + [LINC02057 expression × (0.4507)} + [AC245041.1 expression × (0.1731)} + [AC068228.1 expression × (-0.4206)} + [AL365181.2 expression × (0.1094)}. The correlation heat map also demonstrates the relationship between angiogenesis-related genes and LncRNAs. (Fig. 2E).

**Figure 3.**
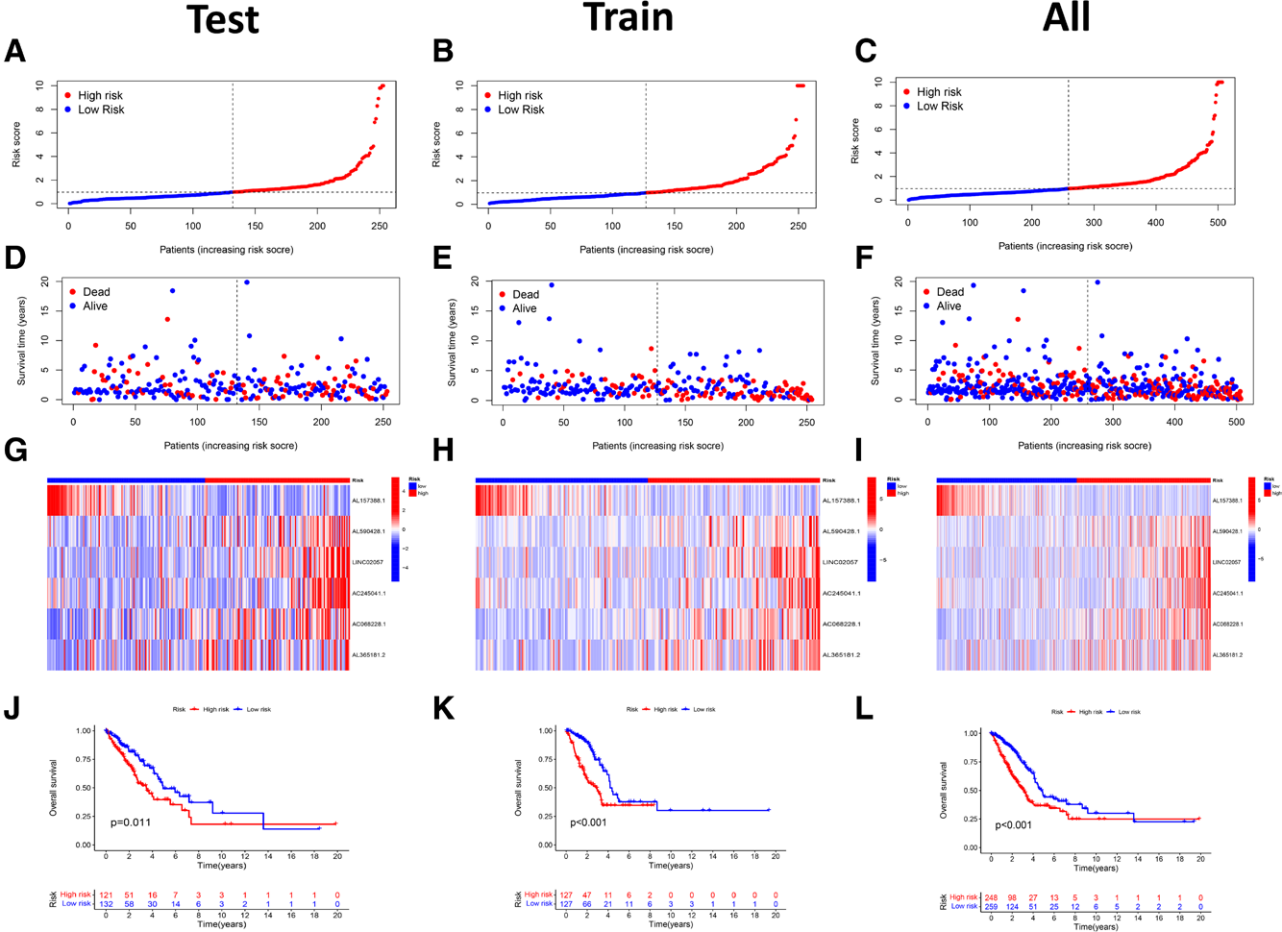
(A–C) Risk scoring curves; (D–F) survival state scatterplot, the green dots represent survival and the red dots represent death; (G–I) heatmap of 6 ARLncRNAs expressions in the high-risk and low-risk groups for the construction of prognostic signature. (J–L) Kaplan–Meier survival curves. ARlncRNAs = angiogenesis-related LncRNAs.

The median risk score was used to stratify LUAD samples into high- and low-risk cohorts. Risk score distribution and patient survival analysis indicated an inverse correlation between risk score and OS in LUAD patients, with those in the high-risk cohort exhibiting a poorer prognosis. Risk score curves and survival status scatter plots were employed to visualize risk stratification and survival outcomes (Fig. 3A–F). Furthermore, heatmap analysis of ARLncRNA expression profiles revealed upregulation of AL590428.1, LINC02057, AC245041.1, AC068228.1, and AL365181.2 in high-risk patients, whereas AL157388.1 expression decreased as the risk score increased (Fig. 3G–I). Kaplan–Meier survival analysis demonstrated that OS was significantly lower in patients with high-risk scores than in patients with low-risk scores (Fig. 3J–L), indicating that risk scores predict OS. We utilized the test cohort and the total cohort for validation to further assess the model’s predictive ability, and the findings were identical to those of the training cohort. These findings confirm the strong prognostic performance of the ARLncRNA-based risk signature.

### 3.3. ARLncRNA as an independent prognostic biomarker for LUAD

**Figure 4.**
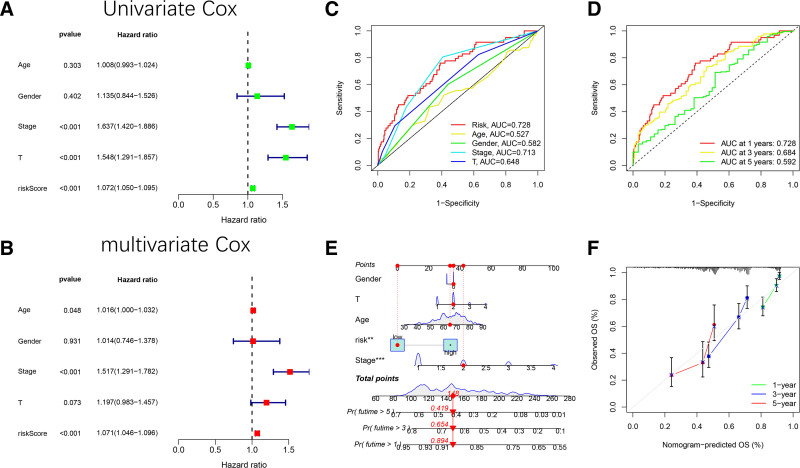
(A) Clinical multi-index ROC curves for the AUC of risk score, age, gender, stage, and T of LUAD patients; (B) time-dependent ROC curves for the AUC of overall survival at 1, 3, and 5 years of LUAD patients; (C) univariate Cox regression analysis; (D) multinomial Cox regression analysis; (E) construction of a nomogram combined clinical indicators and risk score for predicting survival probabilities at 1, 3, and 5 years of LUAD patients; (F) calibration charts for validating the predictive accuracy of the 1-, 3-, and 5-year survival probabilities of the nomogram. AUC = area under the curve, LUAD = lung adenocarcinoma, ROC = receiver operating characteristic.

The constructed ARLncRNA-based risk prognostic model demonstrated its potential as an independent prognostic factor for LUAD, as evidenced by univariate Cox regression analysis, which yielded a hazard ratio (HR) of 1.072 (95% CI: 1.050–1.095, *P* < .001) (Fig. 4A), and multivariate Cox regression analysis, which reported an HR of 1.071 (95% CI: 1.046–1.096, *P* < .001) (Fig. 4B).The predictive accuracy of the prognostic features was assessed using ROC analysis (Fig. 4C–D), indicating that the model outperformed other clinical factors in predicting risk score and survival time. Nomogram survival plots were constructed to predict 1-, 3-, and 5-year survival in patients with LUAD by applying age, gender, stage, T, and risk score factors (Fig. 4E). The 1-, 3-, and 5-year calibration plots were close to the solid gray lines, demonstrating that the nomograms have a high predictive power (Fig. 4F).

### 3.4. PCA and functional enrichments analysis

**Figure 5.**
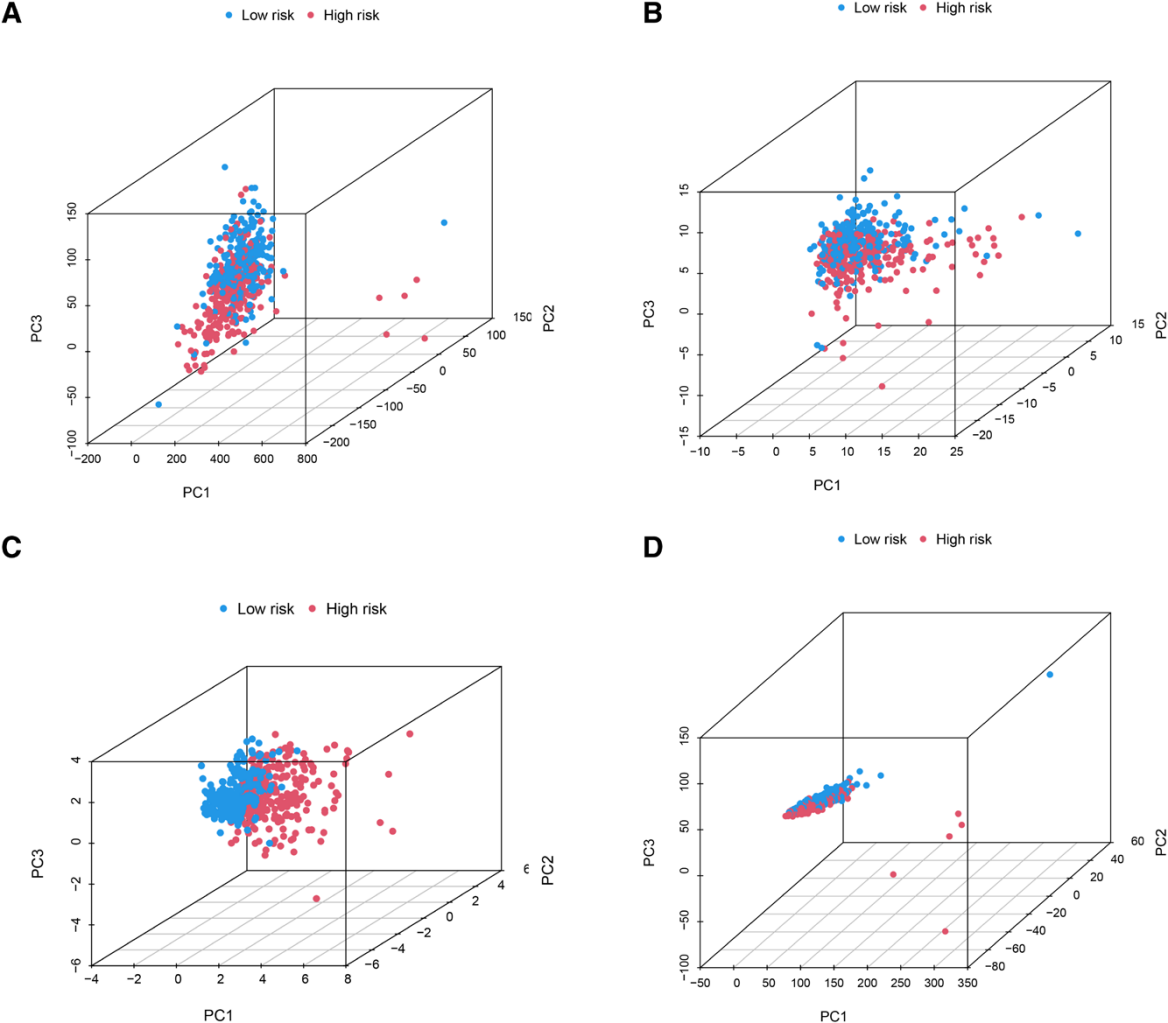
PCA between low and high-risk groups. (A) The all genes set, (B) angiogenesis genes set, (C) ARLncRNAs set, (D) 6 ARLncRNAs set. ARlncRNAs = angiogenesis-related LncRNAs, PCA = principal component analysis.

PCA demonstrated distinct distribution patterns for all genes, angiogenesis-related gene sets, LncRNA gene sets, and ARLncRNA gene sets between the high- and low-risk cohorts (Fig. 5A–D). The findings indicate that LUAD patients could be effectively stratified into 2 cohorts based on the expression of 6 ARLncRNAs, with the high-risk cohort exhibiting a distinct angiogenic profile compared to the low-risk cohort.

### 3.5. Tumor mutation burden analysis

**Figure 6.**
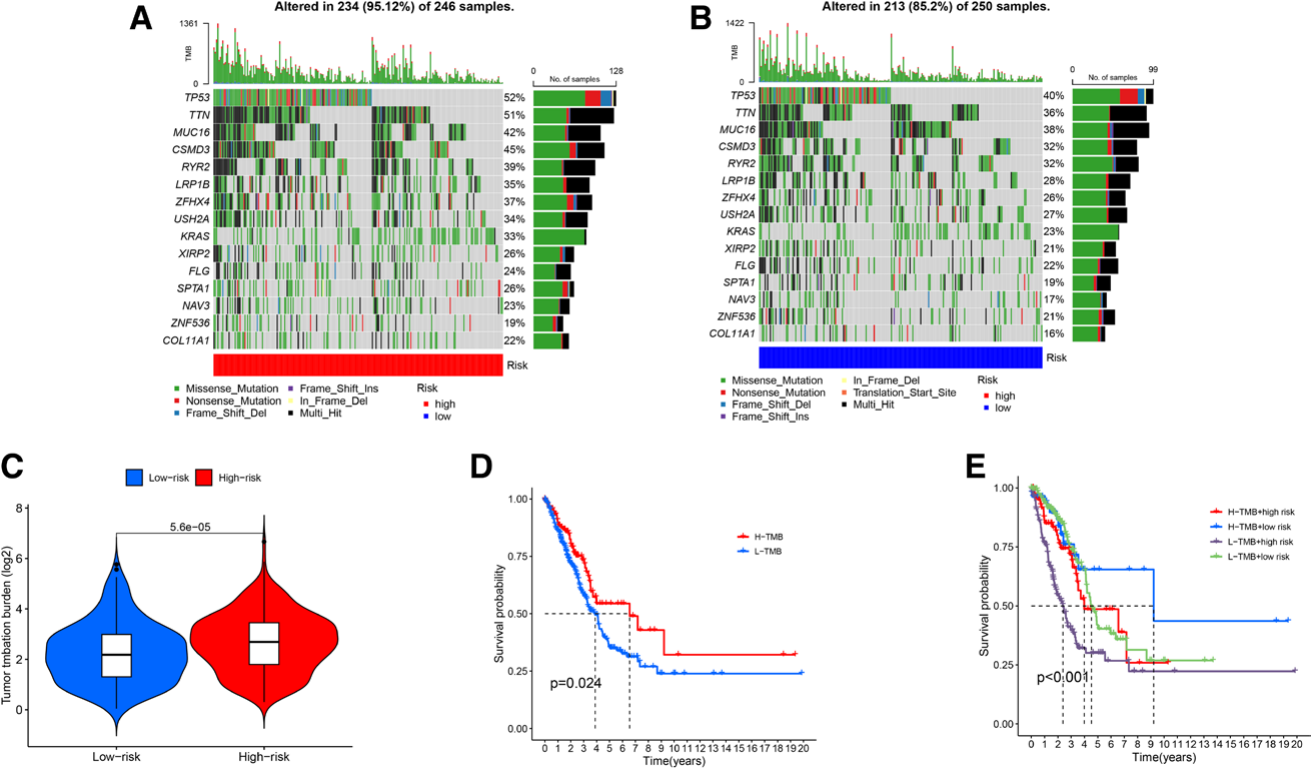
(A–B) Tumor mutation burden analysis of the difference between the high-risk group and low-risk group in LUAD patients. (C) Differential TMB in high-risk and low-risk groups in LUAD. (D) Kaplan–Meier survival analysis of high TMB group (H-TMB) and low-TMB group (L-TMB). (E) Kaplan–Meier survival analysis between the groups of H-TMB + high risk, H-TMB + low risk, L-TMB + high risk, and L-TMB + low risk, Comparison of MST between groups: H-TMB + low-risk group > L-TMB + low-risk group > H-TMB + high-risk group > L-TMB + high-risk group. LUAD = lung adenocarcinoma, MST = median survival time, TMB = tumor mutational burden.

The TMB of LUAD samples from the TCGA database was analyzed to evaluate differences in gene mutations between high- and low-risk cohorts. In the high-risk cohort, the 5 most frequently mutated genes were TP53 (52%), TTN (51%), MUC16 (42%), CSMD3 (45%), and RYR2 (39%), with an overall mutation rate of 95.12% (Fig. 6A).The low-risk cohort had an 85.2% mutation rate, with the top 5 genes having the highest mutation frequency being TP53 (40%), TTN (36%), MUC16 (38%), CSMD3 (32%), and RYR2 (32%) (Fig. 6B). The majority of the alterations in both LUAD samples were missense mutations. We divided the population into high- and low-TMB cohorts based on the median TMB score in order to investigate the prognostic significance of TMB, and then we ran a survival analysis on each cohort. The results revealed a statistically significant difference in TMB levels between the high-risk and low-risk cohorts (*P* < .05, Fig. 6C). More research was done on potential survival differences between patients with high- and low-TMB. The high TMB cohort outlived the low-TMB cohort in terms of survival time (*P* < .05) (Fig. 6D). The high TMB + low-risk cohort had the greatest prognosis, while the low-TMB + high-risk cohort had the poorest prognosis, according to the study of TMB survival and risk score, which revealed a statistically significant difference in median survival time across the 4 cohorts(Fig. 6E).

### 3.6. The ARLncRNA signature of the tumor immune microenvironment and drug screening

**Figure 7.**
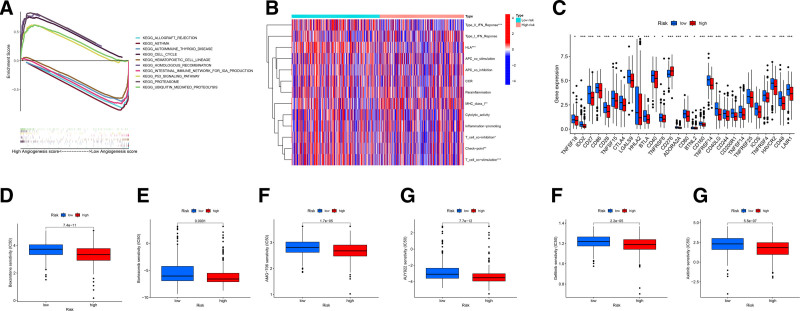
The differences of tumor immune microenvironment between the high- and low-risk cohorts. (A) Gene set enrichment analysis of the top 5 pathways that are significantly enriched in high- and low-risk populations. (B) The difference of immune-related functions in the risk cohorts. (C) The difference of common immune checkpoint expression in the risk cohorts. (D–G) Antiangiogenic drugs sensitivity analysis. LUAD patients with a low -risk score had a higher IC_50_ value of many therapeutic drugs compared with patients with a high-risk score. IC_50_ = half-maximal inhibitory concentration, LUAD = lung adenocarcinoma.

GSEA has the advantage of exploring the signaling pathways involved,^[20]^ and we performed GSEA to explore potential biological functional differences between high- and low-risk cohorts, depending on the prognosis of the patients. GSEA results revealed that ARLncRNA expression was significantly enriched in the high-risk cohort during the immune response (Fig. 7A). Subsequently, risk scores and immune-related functions were analyzed to estimate the immune status of the high- and low-risk cohorts. The results indicated that Type_II_IFN_response, HLA, checkpoint, and T_cell_co-stimulation responses were significantly more active in the low-risk cohort than in the high-risk cohort. In contrast, MHC_class_I responses were significantly more active in the high-risk cohort than in the low-risk cohort. However, no significant differences were observed in other immune functions between the 2 cohorts (*P* > .05, Fig. 7B). It indicated that the immune function of patients in the lower cohort was more active. Additionally, we analyzed immune checkpoint activation across different risk groups and found that nearly all immune checkpoints were more active in the low-risk cohort (Fig. 7C).

The IC_50_ which represents the concentration required to inhibit 50% of cell growth, is widely used to evaluate drug efficacy.^[21]^ At the genetic level, we estimated the IC_50_ values and chemotherapy drug sensitivity, where lower IC_50_ values indicate higher drug sensitivity. That we found that the IC_50_ values of antiangiogenic drugs for lung cancer were lower in the high-risk cohort (Fig. 7D–G), which indicated that patients in the high-risk cohort had higher sensitivity to antiangiogenic drugs.

### 3.7. Expression pattern validation of 6 screened LncRNA

**Figure 8.**
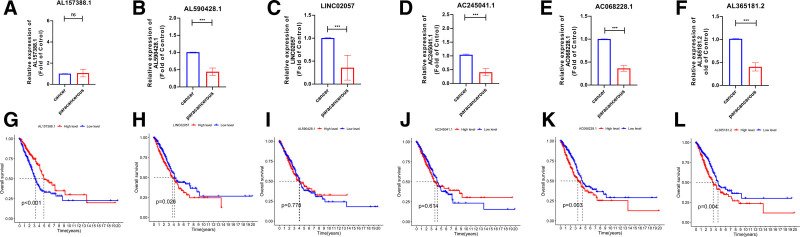
Expression patterns and prognostic values of 6 identified ARLncRNAs. (A–F) Validation of the expression patterns of 5 ARLncRNAs based on 10 cases of lung tumor tissues and their adjacent para-tumor tissues from surgically resected lung patients by qRT-PCR analysis. (G–L) The association of each ARLncRNA’s expression level with Lung cancer patients’ prognosis in the TCGA database. ARlncRNAs = angiogenesis-related LncRNAs, qRT-PCR = quantitative real-time polymerase chain reaction, TCGA = The Cancer Genome Atlas.

The expression levels of AL590428.1, LINC02057, AC245041.1, AC068228.1, and AL365181.2 were verified using qRT-PCR in 16 tumor samples alongside paired normal samples. The results indicated that the expression of these genes was significantly higher in tumor tissues compared to normal tissues (Fig. 8A–F). Kaplan–Meier curves demonstrated that low expression levels of AL157388.1, AL590428.1, AC068228.1, and AL365181.2 were associated with better outcomes, while high expression of AL157388.1 correlated with improved patient prognosis (Fig. 8G–L). However, no statistically significant difference in AL157388.1 expression was observed between tumor and paired normal samples, likely due to the small sample size. These findings highlight the potential of these genes for use in diagnostic and prognostic assessments.

## 4. Discussion

LUAD is a biologically and mechanistically complex malignancy with high morbidity and mortality rates. Low-dose computed tomography is currently an effective screening tool for lung cancer, significantly reducing lung cancer-related mortality. However, its high false-positive rate remains a major limitation.^[22]^ Therefore, developing precise, individualized treatment strategies and establishing a robust prognostic model for accurate disease prediction and risk assessment are of critical clinical importance. Despite the fact that there are numerous markers employing LncRNA to forecast LUAD survival outcomes,^[23–25]^ the LncRNA predictive signature associated with angiogenesis in lung cancer has not been reported. In this study, we were constructed an angiogenesis-related LncRNA marker in order to investigate the relationship with prognosis of LUAD patients. Tumor angiogenesis is a highly intricate and tightly regulated process.^[26]^ We combined the angiogenic gene database with the integrin pathway-related gene database to define angiogenic-related genes in a more comprehensive strategy.^[27]^ Integrins, a family of cell adhesion molecules, play pivotal roles in signal transduction, mechanotransduction, and cell migration, contributing to cancer metastasis. Their crucial involvement in tumor angiogenesis further highlights their potential as therapeutic targets in lung cancer.^[28]^

LncRNA is an essential regulator of tumor markers and also plays a key role by regulating gene expression in the pathogenesis of angiogenesis.^[29]^ An increasing body of research highlights the significant involvement of LncRNAs throughout the angiogenic process in LUAD. LINC00173.v1 facilitates lung cancer angiogenesis by acting as a sponge for miR-511-5p, thereby regulating VEGFA expression.^[30]^ Similarly, LINC00607 interacts with the chromatin remodeling factor BRG1 to sustain ERG target gene transcription, ultimately promoting angiogenesis.^[31]^ However, rather than just 1 LncRNA, a panel of LncRNA biomarkers connected to the stage of the angiogenic process would be more effective. Therefore, we constructed an ARLncRNA-based prognostic model to enhance predictive accuracy and offer novel insights into LUAD diagnosis, treatment strategies, and therapeutic target identification.

In this study, we developed a LUAD risk prediction model using Cox regression analysis and LASSO regression, incorporating 6 ARLncRNAs. Among them, 5 (AL590428.1, LINC02057, AC245041.1, AC068228.1, and AL365181.2) were identified as potential high-risk LncRNAs (HR > 1), while 1 (AL157388.1) was classified as a potentially protective LncRNA (HR < 1). Previous research has demonstrated that AL590428.1 is a cuproptosis-related LncRNA implicated in the prognostic model of bladder cancer, suggesting its potential role in regulating metal ion metabolism within the tumor microenvironment and influencing angiogenesis.^[32]^ Han et al found that AC245041.1 is also an Angiogenesis-related LncRNA to predict the prognosis signature of bladder cancer,^[27]^ which indicates that AC245041.1 is involved in the regulation of multiple tumor angiogenesis. AC068228.1 has also been recognized as a key prognostic biomarker in lung cancer, potentially driving malignant progression by modulating tumor invasion and angiogenesis.^[33]^ Furthermore, AL365181.2 has been repeatedly identified as an immune-related LncRNA associated with LUAD prognosis, suggesting that its role in shaping the tumor immune microenvironment may indirectly influence angiogenesis.^[34,35]^ Although no direct evidence has yet established the involvement of LINC02057 and AL157388.1 in angiogenesis, their inclusion in our prognostic model implies potential functional relevance in angiogenesis-related pathways, warranting further investigation.

The prognostic model was developed using a training cohort to predict OS and mortality. The results demonstrated that patients in the high-risk cohort exhibited significantly lower OS and higher mortality compared to those in the low-risk cohort. These findings were subsequently validated in both the test cohort and the overall cohort. Clinical multi-parameter and time-dependent ROC curves were used to validate the model’s prediction potential for LUAD patients. By using univariate and multiple Cox regression analysis, this model was verified as an independent prognostic factor for LUAD. Nomograms are intuitive and easy to understand, and are suitable for medical research and clinical practice.^[36]^ We generated nomograms utilizing differences in age, sex, stage, and risk score. The calibration plots demonstrated that the predicted 1-, 3-, and 5-year survival rates closely aligned with actual observations, reinforcing the model’s predictive accuracy. By explicitly dividing the samples in the PCA analysis based on the ARLncRNAs prognostic model into a high-risk cohort and a low-risk cohort, it was shown that the vascular status may vary between the 2 cohorts. This suggests that the LUAD prediction model created by the aforementioned 6 ARLncRNAs has strong and accurate predictive capacity, enabling doctors to quickly determine prognosis and individualize initial diagnosis and treatment decisions. Our approach aligns with recent advancements in bioinformatics-driven identification of prognostic LncRNAs. For instance, Bai et al utilized similar correlation and LASSO regression approaches to construct cuproptosis-related LncRNA signatures in bladder cancer.^[37]^ Additionally, Song et al demonstrated that pyroptosis-related LncRNAs could stratify LUAD prognosis.^[38]^ Collectively, these studies, along with our findings, highlight the critical role of pathway-driven LncRNA signatures in precision oncology.

To further assess the clinical applicability of our risk model, we conducted a TMB analysis. Our findings revealed that patients with high TMB exhibited elevated TP53 expression. TP53 is among the most frequently mutated genes in cancer cells, and its mutant forms contribute to tumor progression by enhancing cancer cell proliferation, migration, invasion, metabolic reprogramming, and angiogenesis.^[39]^ The high TMB + low-risk cohort had the best prognosis, whereas the low-TMB + high-risk cohort had the worst, according to the survival analysis coupled with TMB and risk ratings. These results suggest that incorporating TMB into our prognostic model enhances its predictive accuracy and clinical relevance in LUAD. Immunotherapy is the most progressive antitumor therapy that enhances the therapeutic effect by activating immune function.^[40]^ The clinical significance of TMB in predicting the effect of immunotherapy is controversial. In the CheckMate-026 clinical trial, the high- and low-TMB cohorts had no difference in OS between immunotherapy and chemotherapy.^[41]^ With the aim of better evaluating the feasibility of immunotherapy, based on the analysis of the differences in immune-related pathways between the high- and low-risk cohorts, the low-risk cohort was more immunologically active. Furthermore, we examined the relationship between ARLncRNA expression profiles and the expression of immune checkpoint genes. According to several researchers, the effectiveness of immunotherapy is closely associated with the expression levels of immune checkpoint genes.^[42]^ Our findings revealed that low-risk LUAD patients exhibited higher expression levels of key immune checkpoints compared to high-risk patients. This suggests that low-risk LUAD patients may derive greater benefit from immunotherapy, highlighting the potential utility of our model in guiding personalized immunotherapeutic strategies.

For individuals with LUAD, antiangiogenic treatment is a crucial therapeutic option.^[43]^ To identify potential candidates for such therapies, we estimated the IC_50_ of various antiangiogenic drugs and examined the relationship between our risk prognosis model and drug sensitivity. Our findings demonstrated that the high-risk cohort exhibited lower IC50 values for 6 antiangiogenic agents, suggesting that patients in this group may be more responsive to these treatments. As a result, the high-risk population is more eligible for antiangiogenic medication treatment than the low-risk cohort because it is less prone to drug resistance. One potential explanation for this increased sensitivity is the association between TP53 mutations and the VEGF signaling pathway, which may lead to the upregulation of angiogenesis-related processes in the high-risk cohort. This observation provides novel insights into the molecular mechanisms underlying the differential response to antiangiogenic therapy and highlights the potential of our risk model in guiding personalized treatment strategies.^[44]^

Despite its valuable insights, this study has certain limitations. As a retrospective analysis based on publicly available TCGA data, it lacks validation using clinical specimens. Consequently, further experimental studies are required to substantiate our findings and assess their clinical applicability.

## 5. Conclusion

In conclusion, this study is the first to construct a prognostic profile of ARLncRNA associated with survival in LUAD patients whose predictive power has been validated as a stand-alone prognostic indicator. These results offer fresh perspectives on the role of ARLncRNAs in lung cancer and help open up new views for the discovery of potential targets and clinical treatment of LUAD.

## Author contributions

**Conceptualization:** Qing Lei, Zhong-Rui Ma.

**Formal analysis:** Xi-Rui Zhu.

**Funding acquisition:** Wei-Wei Wang.

**Investigation:** Xi-Qi Xie.

**Project administration:** Bo-Wen Cui.

**Resources:** Jin-Xiang Kong.

**Supervision:** Wei-Wei Wang.

**Writing – original draft:** Qing Lei.

**Writing – review & editing:** Biao-Feng Fan.

## Supplementary Material


