# Supplementary material for: Immune infiltration and drug treatment response of angiogenesis-related LncRNA in lung adenocarcinoma
Source: Medicine (Baltimore). 2025 Jul 4;104(27):e42958. doi: 10.1097/MD.0000000000042958 (PMC12237316; doi:10.1097/MD.0000000000042958)
Supplement: Supplementary file 1 [file medi-104-e42958-s001.docx]

AL157388.1 Forword (5’-3’) GCAGGACGGGAGAAGGAAG

Reverse(5’-3’) TGAAGATAGGATTGGCATTGCTT

AL590428.1 Forword (5’-3’) CGGGTTCCTCAGGCTGTTT

Reverse(5’-3’) GTGCGAGTTCTCTTCTTCTTTT

LINC02057 Forword (5’-3’) TTTACCCGTGTTTTTAAGGG

Reverse(5’-3’) GGCTGTCTGTGTATGGTCTGT

AC245041.1 Forword (5’-3’) GAGTGTGTTTTTCCTGCTAA

Reverse(5’-3’) CCTCCCCTACATTCTTTTAT

AC068228.1 Forword (5’-3’) TACCGCTGTCCTGAGCAATG，

Reverse(5’-3’) CCTTCCCGTTTCTCTCTTCCCC

AL365181.2 Forword (5’-3’) AAATGCAGGCGTTGCTG

Reverse(5’-3’) GGGTTTTTATGTGAGGTTCTCA

GAPDH Forword (5’-3’) GGACCTGACCTGCCGTCTAG

Reverse(5’-3’) GTAGCCCAGGATGCCCTTGA
